# Longitudinal Multifrequency MR Elastography Reveals Biomechanical Signatures of Progression in a Translational Liver Tumor Model

**DOI:** 10.1002/advs.77626

**Published:** 2026-09-16

**Authors:** Justus Ramtke, Friederike Hesse, Lasse Noack, Justus Klockner, Yubei He, Yu Liu, Yanglei Wu, Robin Schmidt, Selma Saclier, Salma A. S. Abosabie, Tom Meyer, Richard Ruppel, Nikolaus Berndt, Michael Mülleder, Eyk Schellenberger, Nicola Beindorff, Dominik N. Müller, Ingolf Sack, Jing Guo, Shraga Nahum Goldberg, Lynn Jeanette Savic

**Affiliations:** ^1^ Department of Radiology Charité – Universitätsmedizin Berlin Corporate Member of Freie Universität Berlin and Humboldt‐Universität zu Berlin Berlin Germany; ^2^ Experimental and Clinical Research Center (ECRC) A Cooperation of Charité – Universitätsmedizin Berlin and Max Delbrück Center for Molecular Medicine (MDC) Berlin Germany; ^3^ Berlin Institute of Health at Charité – Universitätsmedizin Berlin Berlin Germany; ^4^ Institute of Experimental Biomedicine University Hospital Würzburg Würzburg Germany; ^5^ Rudolf Virchow Center for Integrative and Translational Bioimaging Julius‐Maximilians‐Universität Würzburg Würzburg Germany; ^6^ Department of Radiology and Biomedical Imaging Yale University School of Medicine New Haven Connecticut USA; ^7^ Department of Molecular Toxicology German Institute of Human Nutrition Potsdam‐Rehbruecke (DlfE) Nuthetal Germany; ^8^ Institute of Biochemistry Core Facility High‐Throughput Mass Spectrometry Charité – Universitätsmedizin Berlin Corporate Member of Freie Universität Berlin and Humboldt‐Universität zu Berlin Berlin Germany; ^9^ Berlin Experimental Radionuclide Imaging Center (BERIC) Charité – Universitätsmedizin Corporate Member of Freie Universität Berlin and Humboldt‐Universität zu Berlin Berlin Germany; ^10^ Max Delbrück Center for Molecular Medicine in the Helmholtz Association Berlin Germany; ^11^ Charité ‐ Universitätsmedizin Berlin Corporate Member of Freie Universität Berlin and Humboldt‐Universität zu Berlin Berlin Germany; ^12^ DZHK (German Centre for Cardiovascular Research) Partner Site Berlin Berlin Germany; ^13^ Goldyne Savad Institute of Gene Therapy and Division of Image‐guided Therapy and Interventional Oncology (S.N.G.) Department of Radiology Hadassah Hebrew University Medical Center Jerusalem Israel; ^14^ Department of Radiology and Nuclear Medicine Otto‐von‐Guericke University Magdeburg Magdeburg Germany

**Keywords:** apparent diffusion coefficient, biomechanical imaging markers, collagen, glycosaminoglycans, liver tumor progression, multifrequency magnetic resonance elastography, new zealand white rabbit, proteoglycans, VX2

## Abstract

Conventional magnetic resonance imaging (MRI) often detects liver tumors after morphologic and vascular changes become apparent, whereas biomechanical alterations may precede visible tumor formation. Here, we evaluate multifrequency magnetic resonance elastography (mMRE)‐derived shear wave speed (SWS) and phase angle (ϕ) covering stiffness and viscosity as non‐invasive biomechanical imaging markers for liver cancer formation and progression in a translational rabbit model. VX2 liver tumor‐bearing New Zealand White rabbits underwent five longitudinal multiparametric MRI scans over 14 days, including mMRE at clinically relevant frequencies on a 3 Tesla scanner. SWS and ϕ maps were compared with apparent diffusion coefficient (ADC) maps, histopathology, and proteomics. Tumor SWS and ϕ increased 2–3 days after implantation and were significantly higher than liver at two weeks (SWS: *p* = .002; ϕ: *p* = .008). Tumor ADC decreased significantly only from days 11–13 onward (day 14: *p* = .02). Histopathology and proteomics demonstrated tumoral enrichment of collagen, proteoglycan, and glycosaminoglycan pathways compared to liver, suggesting matrix remodeling as a key contributor to increased stiffness and viscosity measured by mMRE. The early elevation of SWS and ϕ preceding ADC changes highlights mMRE as a promising tool for detecting tumor‐associated biomechanical remodeling and enabling earlier liver cancer diagnosis and monitoring beyond conventional MRI.

## Graphical abstract

Longitudinal multifrequency magnetic resonance elastography (mMRE) performed on a clinical 3 Tesla magnetic resonance imaging system reveals early biomechanical alterations during liver tumor progression in a translational VX2 model. Changes in tumor stiffness and viscosity precede diffusion‐related alterations and are associated with extracellular matrix remodeling, highlighting the potential of mMRE to advance liver cancer imaging toward biologically informed risk stratification and personalized patient monitoring.

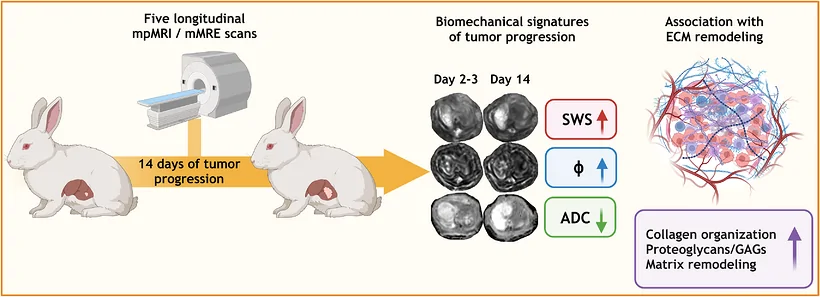

**Abbreviations**
ϕ: phase angle
ADC: apparent diffusion coefficient
DWI: diffusion‐weighted imaging
ECM: extracellular matrix
GAG: glycosaminoglycan
HCC: hepatocellular carcinoma
mMRE: multifrequency magnetic resonance elastography
mpMRI: multiparametric magnetic resonance imaging
PG: proteoglycan
SWS: shear wave speed
TME: tumor microenvironment

## 1 Introduction

Liver cancer is the third leading cause of cancer‐related mortality worldwide, with a rising incidence largely driven by late‐stage diagnosis and the increasing prevalence of chronic liver diseases, particularly metabolic dysfunction–associated liver disease (MASLD) [1]. Current non‐invasive diagnostic approaches rely on characteristic vascular imaging features detected by dynamic contrast‐enhanced magnetic resonance imaging (DCE‐MRI) [2]. However, the imaging appearance of liver tumors is heterogeneous, especially in small or early‐stage lesions, which complicates non‐invasive diagnosis and largely confines imaging to lesion detection rather than enabling assessment of tumor aggressiveness or future growth potential.

Beyond genetic and mutational heterogeneity, the tumor microenvironment (TME) is a key determinant of tumor behavior, influencing cancer cell proliferation, invasion, immune evasion, and resistance to therapy [3]. Central to the TME is the extracellular matrix (ECM) and associated stromal cells, whose dynamic remodeling during carcinogenesis shapes the biomechanical properties of the tumor niche. Specifically, reorganization of ECM components, stromal activation, and angiogenesis, generate mechanical stress that disrupts metabolic flux and leads to changes of interstitial pressure and cellular plasticity [4]. These biomechanical changes not only promote tumor growth and invasion but also establish pro‐tumorigenic niches that act as physical barriers to immune cell infiltration and therapeutic delivery. Consequently, an unmet clinical need exists for imaging approaches that can non‐invasively characterize tumors and their permissive microenvironmental niches before they become morphologically apparent, enabling the stratification of lesions based on aggressiveness and growth potential.

Magnetic resonance elastography (MRE), particularly multifrequency MRE (mMRE), enables non‐invasive assessment of tissue biomechanics by quantifying viscoelastic properties such as stiffness and viscosity through shear wave propagation [5, 6]. Prior studies have demonstrated increased stiffness in liver tumors compared with surrounding parenchyma, underscoring the potential of MRE as a clinical imaging tool for in vivo tumor characterization [7]. However, these investigations have been largely cross‐sectional, leaving the temporal evolution of biomechanical changes during early tumor formation and tumor‐stroma interactions poorly understood.

To address this unmet need, we conducted a longitudinal in vivo study to evaluate the potential of mMRE‐derived imaging biomarkers for early liver tumor detection and to characterize temporal changes in biomechanical properties of liver tumors and their TME over a two‐week period. Tumor progression was serially assessed in a translational VX2 rabbit liver cancer model using a clinical 3T MRI system with mMRE, and imaging findings were corroborated through radiologic‐pathologic correlation and quantitative proteomic analyses of stromal remodeling and ECM composition.

## 2 Methods

### 2.1 Translational Animal Model and Experimental Design

The Berlin State Office for Health and Social Affairs (LAGeSo) approved the animal experiments under the registry number G150/20. Experiments were conducted in accordance with the Federation of European Laboratory Animal Science Associations (FELASA) regulations. Male New Zealand White (NZW) rabbits (2.5–4.0 kg), provided by Charles River Laboratories, were housed in laminar flow rooms under constant temperature and humidity conditions, with ad libitum access to food and water.

Twenty‐three NZW rabbits were used including six donor and 17 recipient rabbits. Liver tumors were induced as previously described [8, 9]. VX2 tumors derived from the donor rabbits were implanted as chunks into the left liver lobes of recipient rabbits and grown for 14 days.

**FIGURE 1:**
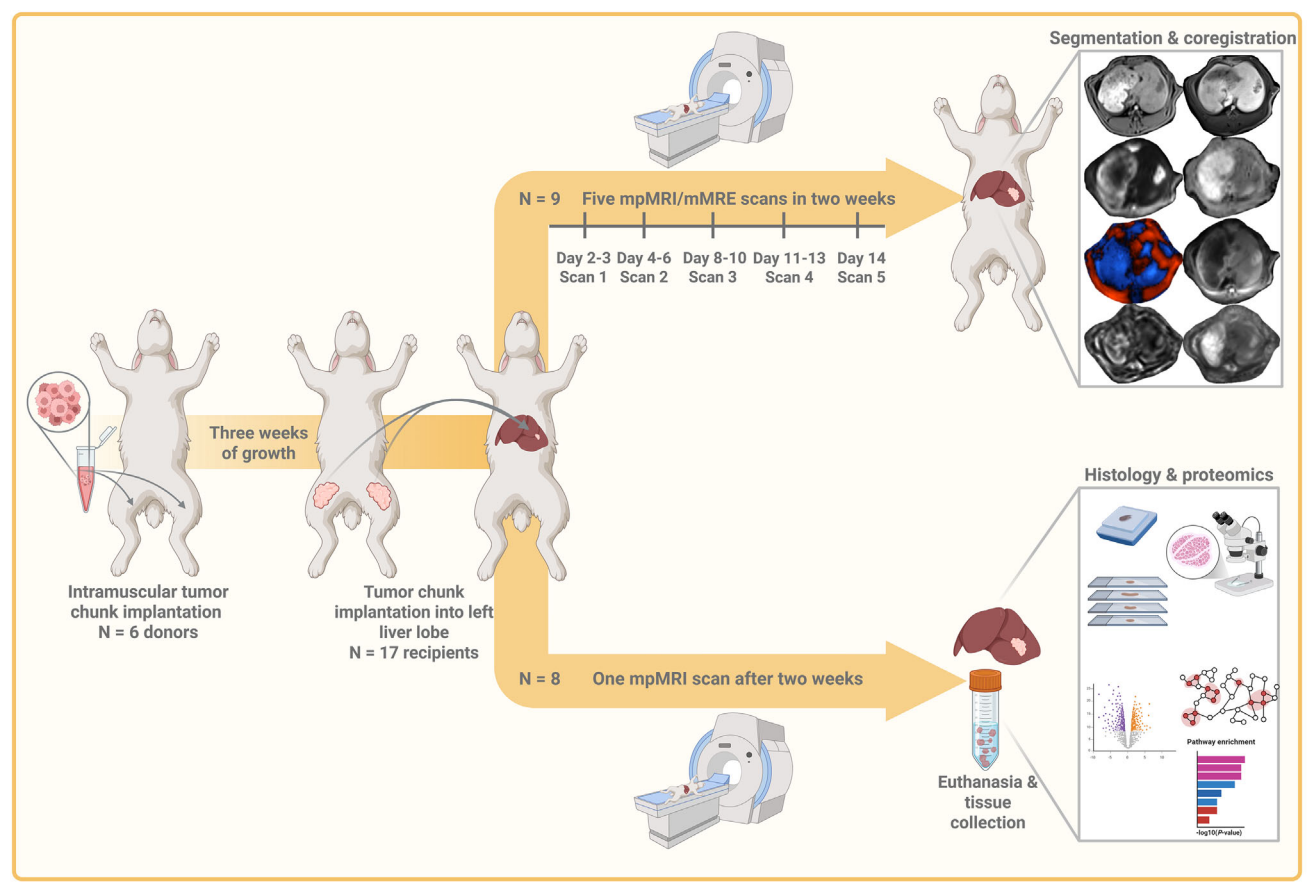
Experimental study design. Twenty‐three rabbits were included. Six served as donors and seventeen as recipients. Recipient rabbits were divided into two arms, where nine underwent five multiparametric MRI / multifrequency MRE (mpMRI/mMRE) scans within 14 days and eight rabbits underwent one mpMRI scan after 14 days, followed by euthanasia. Tissue from this study arm was used for histologic and proteomic analyses.

Nine rabbits underwent five multiparametric MRI (mpMRI)/mMRE scans every 2–3 days for two weeks, resulting in a total of 45 scans. Eight rabbits underwent mpMRI once after 14 days, followed by euthanasia and tissue collection for histopathological and proteomics analysis (Figure 1).

### 2.2 mpMRI Protocols

Imaging of the rabbits was performed using a 3 Tesla MRI scanner (Magnetom Vida, Siemens Healthineers, Germany). Rabbits were scanned inside an 18‐channel inductive transmit‐receive knee coil under general anesthesia using isoflurane gas administered via face mask.

#### 2.2.1 T1‐Weighted Imaging (T1w)

Anatomical reference images were acquired using a T1‐weighted volumetric interpolated breath‐hold examination (VIBE) Dixon sequence with water‐fat separation. The 3D block consisted of 32 slices with an in‐plane resolution of 0.6 × 0.6 mm^2^ and a slice thickness of 2.5 mm. The field of view (FOV) was 248 × 147 mm^2^. The flip angle was 9°, the echo times (TE) were 1.26 and 2.49 ms, the repetition time (TR) was 5.2 ms, and the receiver bandwidth was 965 Hz/pixel.

#### 2.2.2 Multiphasic Contrast‐Enhanced (CE)—MRI

**FIGURE 2:**
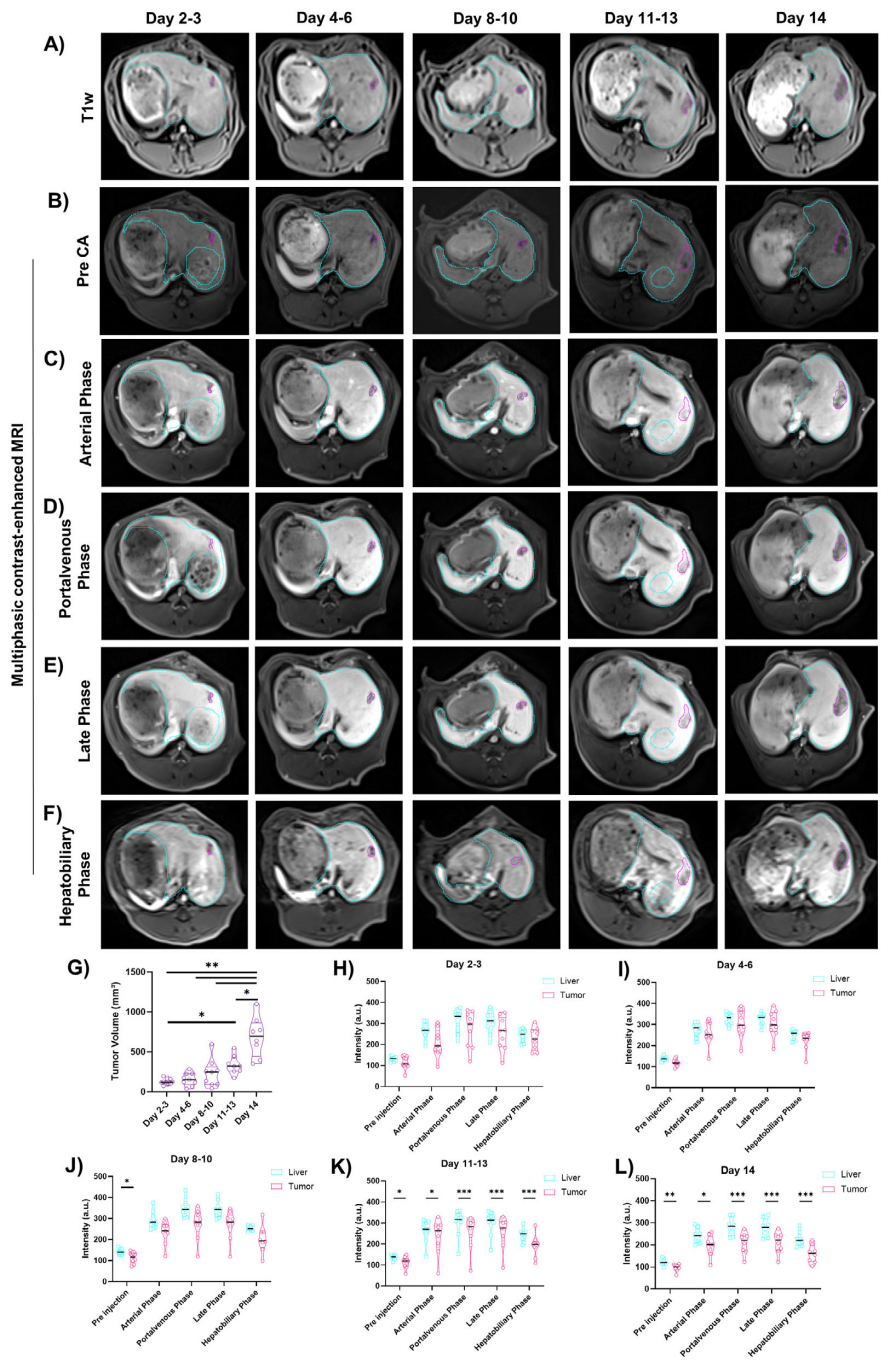
Longitudinal tumor growth and viability. VX2 tumor growth was monitored with five MRI scans over 14 days. Tumors (magenta) and livers (cyan) were segmented and their volume calculated. Multiphasic contrast‐enhanced (CE)‐MRI was performed for monitoring tumor viability by uptake and washout of gadoxetic acid. (A) Longitudinal tumor growth with tumor and liver segmentation masks. (B) T1 vibe GRASP before contrast agent (CA) injection. (C) Arterial phase (D) portalvenous phase (E) late phase (F) hepatobiliary phase (20 min post CA). (G) Tumor volume over time. Quantification of multiphasic CE‐MRI at day 2–3 (H), day 4–6 (I), day 8–10 (J), day 11–13 (K), and day 14 (L). Data are shown as violin plots (*n* = 9) with median, interquartile range, and kernel density estimation, one‐way ANOVA with Tukey‘s multiple comparisons test (tumor growth) and two‐way ANOVA with Sidaks multiple comparisons test (multiphasic CE‐MRI). ^*^ *p*<.05, ^**^ *p*<.01, ^***^ *p*<.001.

For the multiphasic CE‐MRI, gadoxetic acid (Primovist, 0.1 mmol/kg, Bayer, Germany) was injected manually as a bolus injection. Axial imaging was acquired using a golden‐angle radial sparse parallel (GRASP) sequence with a FOV of 199 × 199 mm^2^. The sequence consisted of 40 slices, a slice thickness of 2.5 mm, TE of 1.83 ms, and TR of 4.00 ms. Reconstruction was performed with an in‐plane resolution of 0.9 × 0.9 mm^2^. Seventeen frames were reconstructed with a temporal resolution of 15.6 s from which the arterial, portal venous, and late phases were selected (Figure 2B–E). After performing the multiphasic CE‐MRI, an axial contrast‐enhanced T1 VIBE sequence was acquired 20 min post‐injection to obtain information about the lesion in the hepatobiliary phase (Figure 2F).

#### 2.2.3 Diffusion‐Weighted Imaging (DWI)

DWI was performed using an axial single‐shot spin‐echo echo‐planar imaging (SE‐EPI) sequence with a three‐scan trace approach. The b‐values were 50, 400, and 800 s/mm^2^. The sequence had 12 slices, a FOV of 140 × 140 mm^2^ with an in‐plane resolution of 2.0 × 2.0 mm^2^, and a thickness of 4.0 mm with a 0.8 mm slice gap. TE was 49 ms and TR was 2300 ms. The echo train‐length was 53 and the receiver bandwidth was 1786 Hz/pixel.

#### 2.2.4 Multifrequency MRE (mMRE)

For mMRE acquisition, a belt with three actuators was placed around the rabbit abdomen at the height of the liver. Mechanical vibrations were generated with compressed air using seven different frequencies (40, 50, 60.24, 70.42, 80, 90.09, and 100 Hz) and pressures (350, 400, 450, 500, 550, 600, and 650 mbar). A 2D SE‐EPI sequence with trapezoidal, flow‐compensated motion‐encoding gradients (MEGs), sequentially applied along all three axes of the scanner coordinate system, was used to acquire the 3D wavefield. Eight temporal dynamics per vibration cycle were acquired for each vibration frequency. Thirteen consecutive axial slices were acquired with an in‐plane resolution of 1.6 × 1.6 mm^2^ and a slice thickness of 5.0 mm. Each image was acquired four times and averaged to improve the signal‐to‐noise ratio (SNR). The remaining imaging parameters were as follows: TR, 1090 ms; TE, 40 ms; FOV, 250 × 109 mm^2^; parallel imaging using GRAPPA with an acceleration factor of 2; MEG frequency, 89.93 Hz, resulting in fractional motion encoding for all drive frequencies, providing a practical compromise between motion sensitivity and a short TE. The MEG amplitude was 40 mT/m.

### 2.3 MRE Postprocessing

MRE data were processed using the wavenumber‐based multifrequency dual elasto‐visco inversion (*k*‐MDEV) technique to generate maps of shear wave speed (SWS, m/s) as a surrogate for tissue stiffness [10]. Additionally, multifrequency direct inversion (MDEV) was performed to derive maps of the phase angle (ϕ, rad) which represents tissue viscosity [11, 12]. *k*‐MDEV was selected for SWS because its reliance on first‐order spatial gradients provides robustness against noise and wave‐field imperfections [10], whereas MDEV was selected for ϕ because it directly derives the loss angle of the complex shear modulus [13]. The value of SWS is proportional to the square root of the storage modulus, whereas ϕ ranges from 0 (pure elastic behavior) to π/2 (viscous fluid properties). SWS and ϕ are used to provide quantitative information, while the terms “stiffness” and “viscosity” are reserved for discussing qualitative changes in mechanical properties. MRE data processing was performed using a publicly available online tool [14].

### 2.4 Analysis of MRI/DWI/MRE Data

Three‐dimensional tumor and liver segmentations were performed on water‐only T1w‐Dixon and GRASP sequence images (Figure S1). After segmentation, T1w‐Dixon images were coregistered to both the DWI and the MRE magnitude maps using a rigid‐body transformation with six degrees of freedom (6 DOF). Then, transformation of the segmentation masks to the DWI/ADC‐ and MRE maps was performed to calculate scalar means. Segmentation, coregistration, transformation, and calculation of scalar means were performed using 3D Slicer (version 5.6.1, RRID:SCR_005619) [15]. Both DWI/ADC and mMRE measurements were reviewed in consensus by two radiologists to ensure consistency.

### 2.5 Histopathology and Immunohistochemistry (IHC)

Rabbits were euthanized after the final scan under deep anesthesia by injecting 100 mg/kg Euthadorm (CP‐Pharma, Germany) intravenously. Tissues were harvested, cut into 3–5 mm slices, and immediately fixed in 4% formaldehyde (Carl Roth, Germany) for 24 h. Then, samples were dehydrated in an ethanol series followed by xylene and afterwards embedded into paraffin within 24 h. Tissues were cut into 5 µm thin slices using a Thermo Rotary microtome (Thermo Fisher Scientific, USA). Sections were then stained with hematoxylin and eosin (H&E) (Carl Roth, Germany) for identification of tumor and liver tissue. Pentachrome (Doello) and Alcian blue stainings were performed to assess collagen, proteoglycan (PG) and glycosaminoglycan (GAG) content [16, 17]. IHC of proliferating cell nuclear antigen (PCNA) was performed with a mouse and rabbit specific HRP/DAB detection IHC kit (AB64264, Abcam Limited, UK) and a PCNA monoclonal antibody (clone PC10, MA5‐11355, Thermo Fisher Scientific, USA, RRID: AB_10984046) at 1:200 dilution. Analysis of histopathological stainings was performed with QuPath (version 0.6.0, RRID:SCR_018257) [18].

### 2.6 Proteomics

Paraffin embedded tissue of VX2 tumors and livers from eight rabbits was cut into 20 µm sections following paraffin removal. Approximately 1.5 mg of tissue was transferred into test‐specific tubes. Analysis was performed using nano‐liquid chromatography (UltiMate 3000, Thermo Fisher Scientific, USA) coupled to a Q Exactive HF Orbitrap mass spectrometer. Peptide separation was accomplished using a 55 cm C18 column and a 121 min gradient. Full scan mass spectra were recorded at a resolution of 60 000, following higher‐energy collisional dissociation fragmentation of the 12 most intense precursor ions. Trypsin digestion and carbamidomethylation as fixed modifications and N‐terminal acetylation and methionine oxidation as variable modifications were performed. Data processing was performed using MaxQuant based upon the mouse UniProtKB database.

Proteomics data were analyzed using a log2 fold‐change for identifying significantly downregulated and upregulated proteins at thresholds of ≤ ‐1 and ≥ 1, respectively. Upregulated proteins were subject to STRING‐dB analysis of protein‐protein interaction, functional enrichment, and to identify proteins associated with ECM [19, 20]. The functional enrichment terms provided by STRING‐dB (RRID:SCR_005223) were then examined to identify proteins within the clusters that are associated with ECM. A subset of 50 ECM‐associated proteins was selected by iteratively compiling proteins that were repeatedly associated with ECM‐related terms. For cluster‐specific functional enrichment analysis, only STRING‐dB clusters containing at least five proteins were considered. Pathway enrichment analysis was performed on this identified subset using the databases Reactome 2024, WikiPathways 2024 Human, and KEGG 2026 in the Enrichr platform (RRID:SCR_001575) to identify related pathways based upon overrepresented signaling [21, 22].

### 2.7 Statistical Analysis

Statistical analysis was performed with GraphPad Prism (version 10.4.1, RRID:SCR_002798). Shapiro‐Wilk was used to test for normality and log‐normality. Tumor growth was evaluated over time using paired one‐way analysis of variance (ANOVA) with Tukey's multiple comparisons test. Intensity between livers and tumors in multiphasic CE‐MRI was analyzed using paired two‐way ANOVA with Sidaks multiple comparisons test. SWS, ϕ, and DWI/ADC were statistically analyzed by paired two‐way ANOVA or mixed‐effects model with Tukey's multiple comparisons test, respectively. Effect size calculations were performed with the Cohen's *d_z_* where values of ±0.2, 0.5, and 0.8 indicate small, medium, and large effects [23]. Pearson and Spearman correlations were performed for normally and log‐normally distributed datasets, respectively. Histological sections were compared using a mixed‐effects model with Tukey's multiple comparisons test. *p*<.05 was considered statistically significant.

## 3 Results

### 3.1 Longitudinal Liver Tumor Growth

Liver tumor induction was successful in all animals. Tumor growth was observed from day 2–3 imaging with an increase in size up to day 14 (Figure 2A,G). Two weeks post inoculation, the tumors reached a volume of 687.39 ± 249.51 mm^3^ compared to the starting volume of 124.49 ± 36 mm^3^ (*p* = .003).

### 3.2 Tumor Viability and Cellularity Assessed by Multiphasic CE‐MRI, ADC, and IHC of PCNA

**FIGURE 3:**
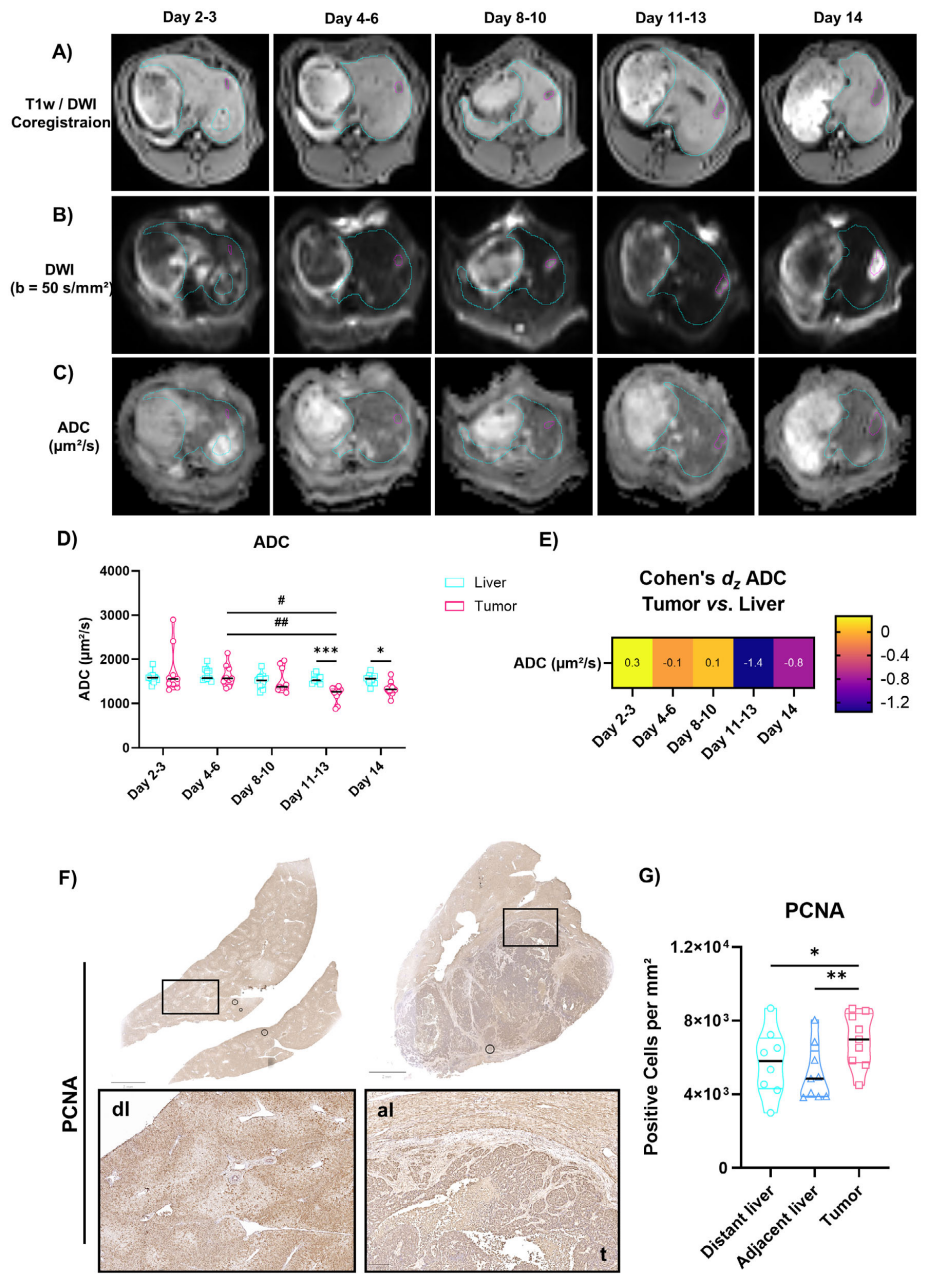
Tumors exhibit increased cellular density and proliferation over time. (A) Rigid coregistration of T1w images to DWI space. Representative images of DWI (B) and ADC (C) with segmentation masks for tumors (magenta) and livers (cyan) after body transformation. (D) Quantification of ADC for liver and tumor. (E) Heatmap of Cohen‘s *d_z_* ADC between liver and tumor. (F) Immunohistochemistry of proliferating cell nuclear antigen (PCNA) expression in distant liver (dl, left) and tumor with adjacent liver (t, al, right). (G) Quantification of PCNA positive cells per mm^2^ in the distant liver, adjacent liver, and tumor. Data are shown as violin plot (*n* = 9) with median, interquartile range, and kernel density estimation, mixed‐effects model with Tukey‘s multiple comparisons test. ^*^ *p*<.05, ^**^ *p*<.01, ^***^ *p*<.001, ^#^ *p*<.05, ^##^ *p*<.01. * = comparison between liver & tumor, # = comparison between tumors.

Tumors exhibited no significant changes in contrast uptake between days 2–10, while only showing hypointense trends (Figure 2H–J). By 11–13 days, tumor hypointensity became statistically significant compared to the liver on the multiphasic CE‐scans (Figure 2K). At day 14, tumors exhibited an intensity of 159.68 ± 40.15 a.u. while liver intensity was 225.57 ± 32.64 a.u. in the hepatobiliary phase (*p*<.001, Figure 2L). Similarly, over days 2–10 ADC of tumors declined over time compared to livers with becoming significant by days 11–13 (Figure 3A–D). At day 14, tumor ADC was 1343 ± 175 µm^2^/s while liver ADC was 1552 ± 127 µm^2^/s, *p* = .02, and an effect size of *d_z_*  =  0.8 (Figure 3C–E) indicating restricted diffusion due to higher cellularity of tumors. IHC stainings of PCNA confirmed increased cell proliferation in tumor areas (t) compared to the adjacent liver (al) and distant liver (dl) (t: 6993 ± 1443 vs. al: 5139 ± 1500 PCNA positive cells/mm^2^, *p*<.01, and vs. dl: 5725 ± 1820 PCNA positive cells/mm^2^, *p*<.05, Figure 3F,G). Furthermore, H&E staining showed higher cell density in tumors than in liver parenchyma (t: 9628 ± 1168 vs. al: 7150 ± 3143 cell nuclei/mm^2^, *p*<.01, and t: 9628 ± 1168 vs. dl: 7247 ± 2900 cell nuclei/mm^2^, *p*<.05, Figure 6A–C).

### 3.3 MRE‐Derived Tumor Stiffness and Viscosity

**FIGURE 4:**
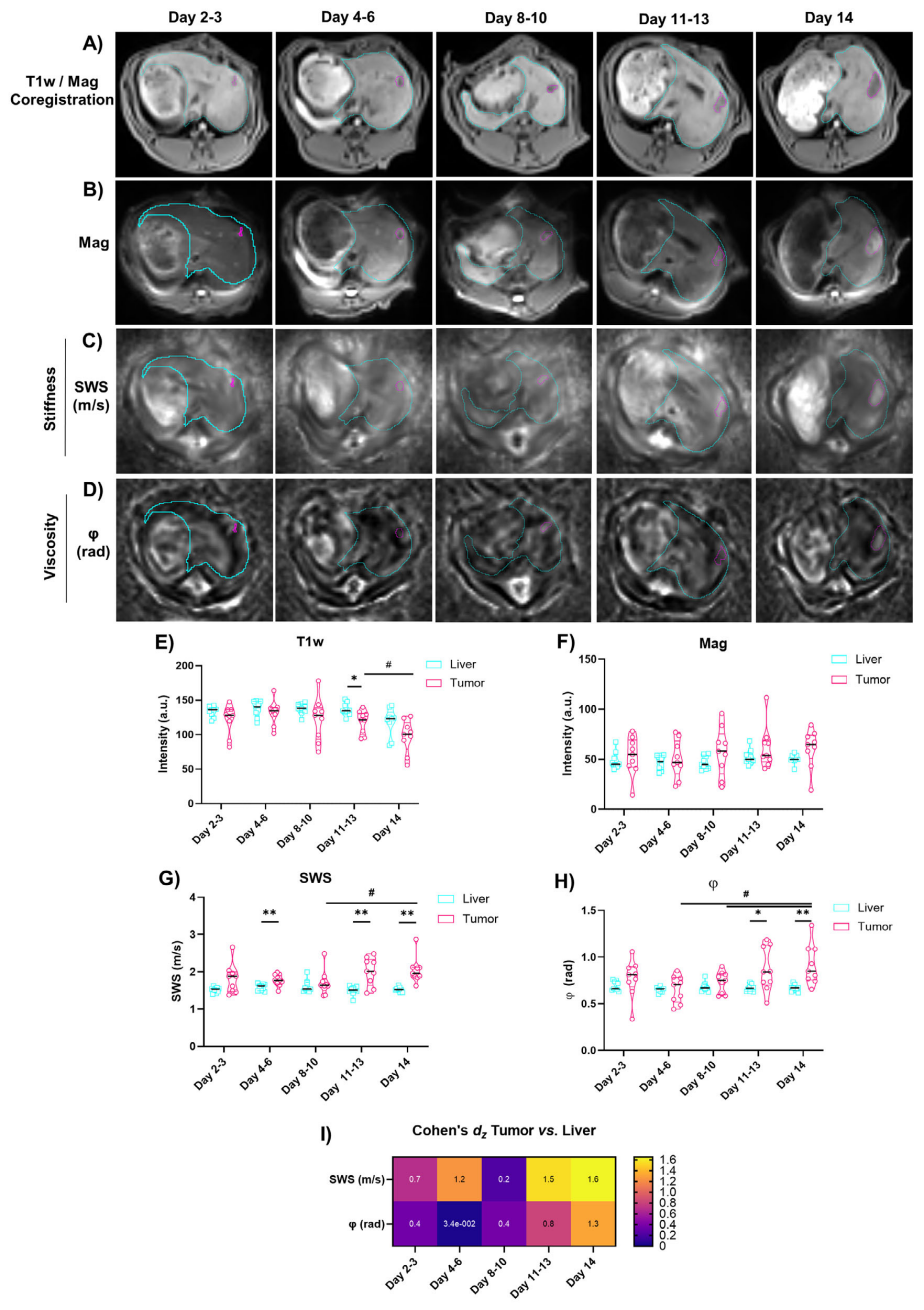
Stiffness and viscosity are increased during tumor growth. (A) Rigid coregistration of T1w images with MRE maps. T1w images and segmentation masks for tumors (magenta) and livers (cyan) after transfer into MRE space using magnitudes as fixed and T1w images as moving images. (B–D) Representative images of MRE magnitude, SWS, and ϕ after adjusted segmentation masks to body registration. (E) Longitudinal quantification of T1w, (F) magnitude (Mag), (G) shear wave speed (SWS), and (H) phase angle (ϕ) for livers and tumors. (I) Heatmap of Cohen‘s *d_z_* MRE SWS and ϕ values between tumor and liver for each timepoint. Data are shown as violin plots (*n* = 9) with median, interquartile range, and kernel density estimation, paired two‐way ANOVA with Tukey‘s multiple comparisons test. ^*^ *p*<.05, ^**^ *p*<.01 (comparison between livers and tumors), ^#^ *p*<.05 (comparison between livers or tumors).

Tumor SWS showed an increase during days 2–6 after implantation, with a significant difference between tumor and liver observed at days 4–6. This difference was transiently reduced at days 8–10, before tumor SWS again became significantly higher than liver during days 11–13. At day 14, tumor SWS was 2.04 ± 0.35 m/s compared with 1.52 ± 0.06 m/s in liver (*p* = .002, *d_z_*  =  1.6; Figure 4C,G,I). Tumor ϕ gradually increased over the first three imaging time points (days 2–10) and became significantly higher than liver during days 11–13. At day 14, tumor ϕ was 0.92 ± 0.22 rad compared with 0.67 ± 0.04 rad in liver (*p* = .008, *d_z_*  =  1.3; Figure 4D,H,I). Representative T1w/magnitude coregistration images and corresponding signal intensities are shown in Figure 4A,B,E,F.

### 3.4 Correlation of MRE and ADC Parameters

**FIGURE 5:**
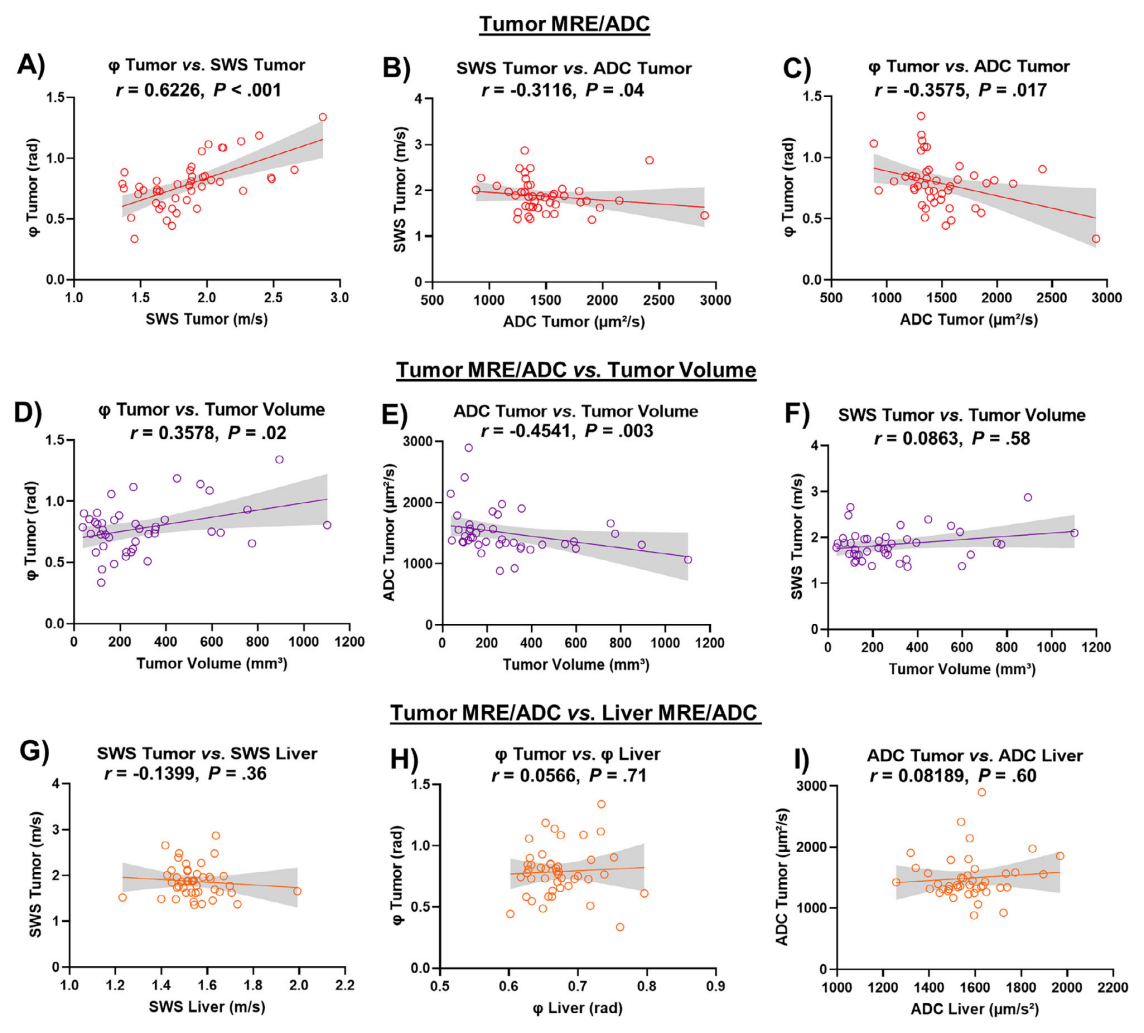
Biomechanical correlations of VX2 liver tumors. (A–C) Correlation of tumor shear wave speed (SWS), phase angle (ϕ), and apparent diffusion coefficient (ADC). (D–F) Correlation of tumor MRE/ADC with tumor volume. (G–I) Correlation of tumor MRE/ADC with liver MRE/ADC. Pearson or Spearman correlation was performed based on the distribution. Pearson or Spearman *r* values are indicated for each dataset and the resulting *p‐*values.

When correlating between stiffness, viscosity, cellularity, and tumor volume (Figure 5), tumor ϕ and tumor SWS showed a positive correlation (*r*  =  0.6226, *p*<.001, Figure 5A). Tumor SWS and tumor ϕ negatively correlated with tumor ADC (*r*  = −0.3116, *p* = .04, Figure 5B) and (*r*  = −0.3575, *p* = .02, Figure 5C). Tumor ϕ showed a positive correlation with tumor volume (*r*  =  0.3578, *p* = .02, Figure 5D), while tumor ADC negatively correlated with tumor volume due to the increased cellularity (*r*  = −0.4541, *p* = .003, Figure 5E). Tumor SWS, however, did not correlate with tumor volume (*r*  =  0.0863, *p* = .58, Figure 5F). When comparing the tumor stiffness, viscosity, and cellularity with those measured in livers, no correlations were found (Figure 5G–I).

### 3.5 Histopathological Analysis of ECM Composition and Stromal Remodeling

**FIGURE 6:**
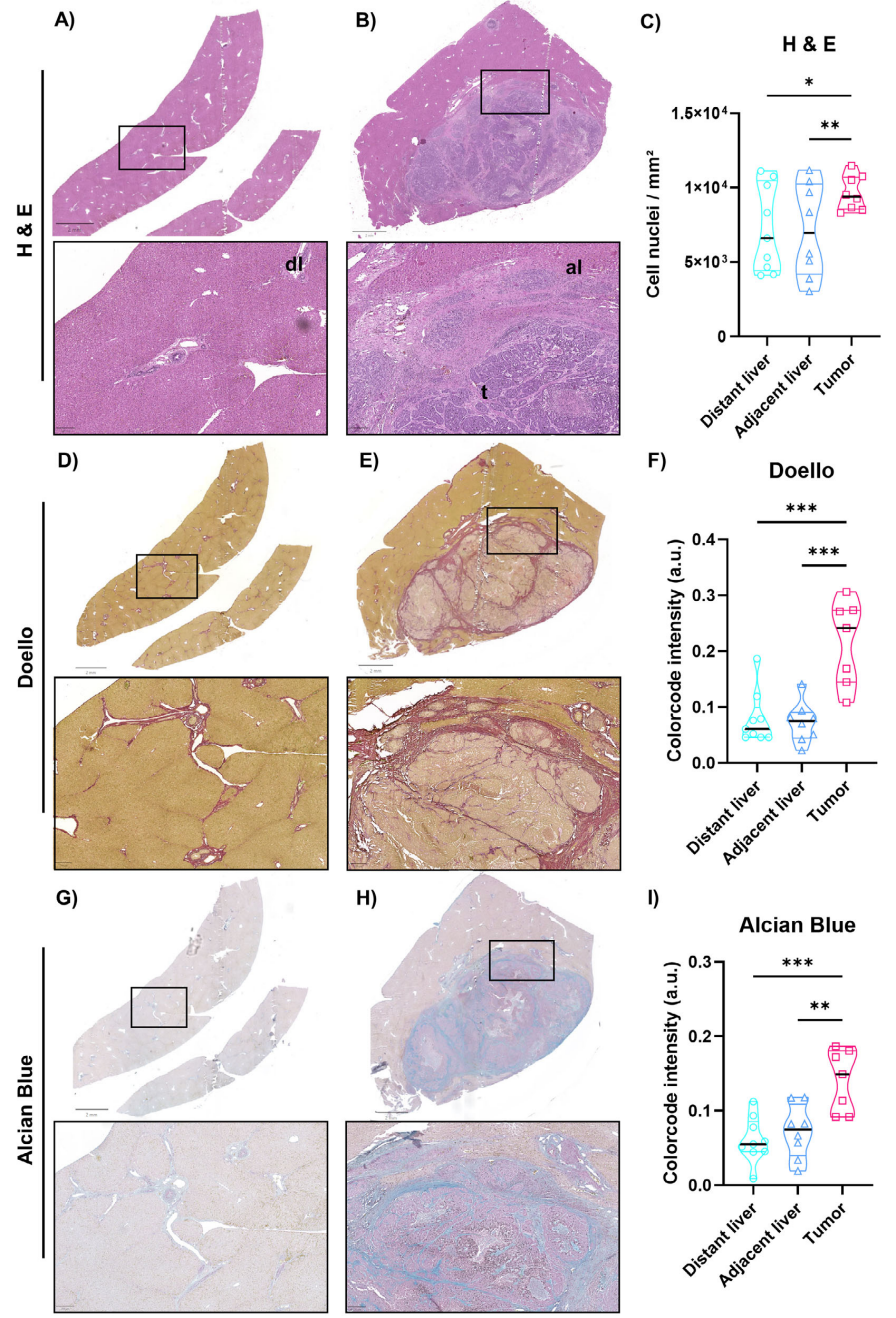
VX2 tumors exhibit increased levels of collagen, proteoglycans, and glycosaminoglycans in their extracellular matrix. (A) Representative images of hematoxylin & eosin (H&E) stained distant liver (dl) and VX2 tumor (t) with adjacent liver (al) (B). (C) Quantification of cell nuclei in distant liver, adjacent liver, and tumor. (D) Doello staining of distant liver and VX2 tumor with adjacent liver (E). (F) Quantification of collagen, proteoglycan, and glycosaminoglycan content in distant liver, adjacent liver, and VX2 tumor. (G) Alcian blue staining of distant liver and VX2 with adjacent liver (H). (I) Quantification of sulfated and carboxylated glycosaminoglycan in distant liver, adjacent liver, and tumor. Data are shown as violin plots with median, interquartile range, and kernel density estimation (*n* = 7–9), mixed‐effects model with Tukey‘s multiple comparisons test. ^*^ *p*<.05, ^**^ *p*<.01, ^***^ *p*<.001.

H & E staining of VX2 tumors (Figure 6B) showed a dense area of cells with necrotic cores that differed from both the adjacent liver parenchyma and the distant liver parenchyma from the right liver lobe (Figure 6A–C). Doello staining of VX2 tumors revealed collagen, PG and GAG rich regions of the ECM in and around the tumor that were significantly different to the adjacent liver (0.216 ± 0.075 a.u. vs. 0.073 ± 0.036 a.u., *p* = .001) and distant liver (vs. 0.079 ± 0.047 a.u., *p* = .001; Figure 6D–F). Alcian blue histopathology (Figure 6G–I) demonstrated tumor ECM exhibiting increased amounts of sulfated and carboxylated GAGs that differed significantly from the adjacent liver (0.141 ± 0.041 a.u. vs. 0.072 ± 0.036 a.u., *p* = .003) and distant liver (vs. 0.061 ± 0.030 a.u., *p* = .001).

### 3.6 Proteomic Analysis for ECM‐Related Pathways

**FIGURE 7:**
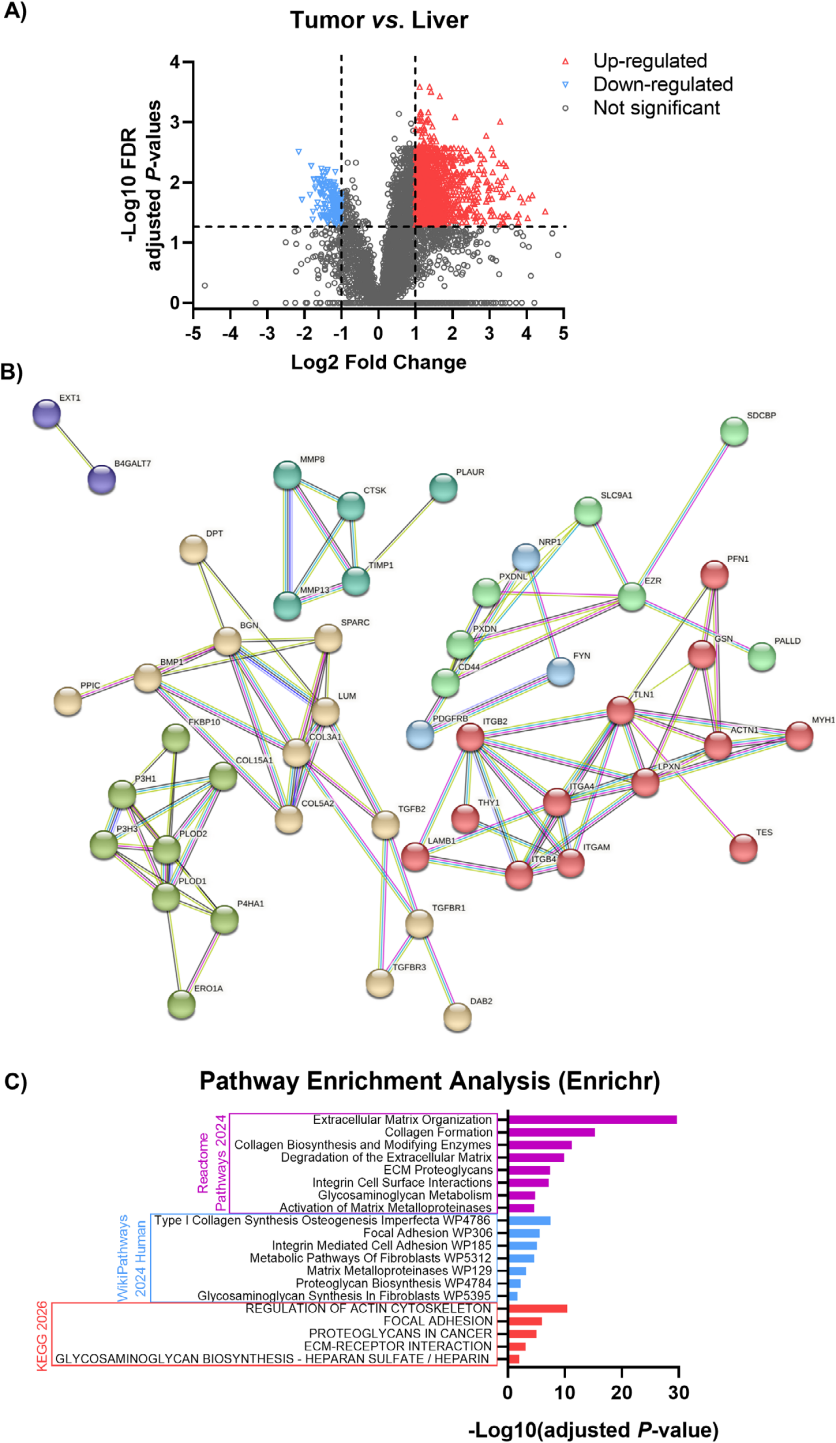
Proteomic characterization of the tumor extracellular matrix (ECM). (A) Volcano plot of tumor vs. liver with significantly upregulated proteins (log2 fold change ≥ 1, red) and significantly downregulated proteins (log2 fold change ≤ ‐1, blue). (B) STRING analysis of upregulated proteins in seven clusters that are associated with ECM pathways. Primary cluster descriptions are as follows: 1/red: Integrin‐mediated signaling pathway, 2/brown: Collagen fibril organization, 3/olive: Protein hydroxylation, 4/green: Hyaluronan uptake and degradation, 5/blue: Activation of matrix metalloproteinases, 6/light sky blue: Angiogenesis involved in coronary vascular morphogenesis, 7/medium blue: Heparin biosynthesis. (C) ‐Log10 of adjusted *p*‐values from 20 pathways that are significantly enriched in the Reactome 2024, WikiPathways 2024 Human, and KEGG 2026 databases with ECM association.

Proteomics analysis identified a total of 5029 proteins that were found in both tumor and liver tissue and were therefore included in the analysis. After setting the log2 fold change thresholds at ≥ 1 for upregulated proteins and ≤ −1 for downregulated proteins and considering an adjusted *p*‐value < 0.05 as significant, 1128 proteins were significantly upregulated, and 155 proteins were significantly downregulated (Figure 7A). STRING‐dB analysis revealed seven clusters with functional and physical connections to ECM‐associated proteins [24]. The clusters were associated with integrin‐mediated signaling pathway (*n* = 13), collagen fibril organization (*n* = 12), protein hydroxylation (*n* = 8), hyaluronan uptake and degradation (*n* = 7), activation of matrix metalloproteinases (*n* = 5), angiogenesis involved in coronary vascular morphogenesis (*n* = 3), and heparin biosynthesis (*n* = 2) (Figure 7B and Table S1). Clusters containing at least five proteins were further considered for cluster‐specific functional enrichment analysis (Table S2). Pathway enrichment analysis (Enrichr) with the libraries Reactome Pathways 2024, WikiPathways 2024 Human, and KEGG 2026 further demonstrated significant enrichment of multiple ECM‐related pathways. Pathways related to collagen formation and biosynthesis, proteoglycans and glycosaminoglycan metabolism or biosynthesis, as well as integrin signaling, focal adhesion, and regulation of the actin cytoskeleton were significantly enriched. A subset of 20 enriched pathways across all three libraries is shown in Figure 7C, while all significantly enriched pathways are shown in Tables S3–S5.

## 4 Discussion

This longitudinal translational study demonstrates that multifrequency MR elastography–derived stiffness and viscosity provide sensitive, noninvasive imaging biomarkers of liver tumor progression under clinically relevant imaging conditions. Using serial mMRE on a clinical 3T MRI system, we show that tumor shear wave speed (SWS) and phase angle (ϕ) increased as early as 2–3 days after tumor implantation and were significantly higher than liver parenchyma at two weeks (SWS *p* = .002; ϕ *p* = .008). By integrating imaging with histopathology and quantitative proteomics, we further linked in vivo imaging findings with elevated collagen, proteoglycan, and glycosaminoglycan content and enrichment of extracellular matrix (ECM)‐associated pathways in liver tumors. Together, these early and dynamic biomechanical alterations enabled clear differentiation of tumors from surrounding liver parenchyma and evolved over time, reflecting progressive remodeling of the tumor microenvironment (TME) during tumor growth. Notably, these biomechanical changes preceded alterations in diffusion‐derived cellularity, as apparent diffusion coefficient (ADC) values remained comparable between tumor and liver during the first week and decreased only at later stages. Thus, the results support mMRE as a non‐invasive tool for early detection and longitudinal characterization of liver tumors beyond conventional MRI.

Increased tumor stiffness measured by MRE has been reported in patient studies and is consistent with our findings in the VX2 model [25, 26, 27]. In this study, SWS increased at early growth stages, while a transient decrease between days 8–10 indicates a non‐monotonic evolution of tumor stiffness during progression. Previous MRE studies have demonstrated associations between tumor stiffness and histopathological characteristics in untreated hepatocellular carcinoma (HCC) [26, 27], suggesting that mechanical properties reflect tumor composition rather than tumor growth alone. VX2 progression is characterized by intratumoral heterogeneity, including necrosis and changes in vascularization with increasing tumor size [28]. Changes in the relative contribution of these tumor compartments may therefore contribute to temporal variability in SWS and could partly account for the transient decrease observed at days 8–10. Tumor ϕ gradually increased during early growth (2–10 days) and remained elevated at later stages (11–14 days), showing a positive correlation with tumor volume and an inverse correlation with ADC, indicating increasing cellularity with tumor expansion. Elevated ϕ has also been described in proliferative HCC and may reflect altered vascularization and vessel density [27, 29]. Additionally, the positive correlation between SWS and ϕ indicates the coupled evolution of stiffness and viscosity, whereas their negative correlations with ADC suggest that increased biomechanical parameters are associated with restricted diffusion and increased cellularity. Notably, SWS did not correlate with tumor volume, suggesting sensitivity to tissue alterations beyond macroscopic growth. Although not statistically significant at every time point, early increases in SWS (days 2–6) suggest that stiffness may be particularly responsive to microstructural tissue changes during initial tumor progression, potentially preceding changes in viscosity and diffusion metrics.

Histopathology revealed increased cellular density, proliferation, and necrosis in VX2 tumors, providing a structural correlate for the restricted water diffusion observed on ADC maps. Consistent with elevated mMRE‐derived stiffness and viscosity, tumors showed increased collagen, PG, and GAG content, indicating pronounced ECM remodeling at both histopathologic and proteomic levels [16, 30]. Notably, proteomic analysis identified enrichment of pathways related to collagen formation and biosynthesis, as well as PG and GAG metabolism and biosynthesis in independent libraries. Collagen is a major structural component of the TME, and alterations in collagen organization are closely linked to mechanical properties of tumor tissue [31, 32]. The activation of matrix metalloproteinases further supports active ECM remodeling, consistent with their role in ECM turnover and degradation [33]. Dysregulated GAG and PG metabolism has been linked to mechano‐transduction and tumor progression in previous studies [34]. Specifically, heparan sulfate (HS) GAGs have been reported to play a role in HCC vascularization and growth [35, 36]. On a molecular level, HS GAGs regulate receptor signaling, while carboxylated GAGs influence water binding and interstitial transport [37, 38]. Furthermore, increased integrin signaling, focal adhesion, and regulation of the actin cytoskeleton suggest altered cell‐ECM interactions, as well as altered mechanotransduction within the TME [31, 32]. These molecular and structural changes are expected to alter physiological transport properties and tissue mechanics, thereby affecting mMRE measurements, DWI, and contrast‐agent kinetics, respectively. Hence, they provide a mechanistic basis for the observed increased stiffness, viscosity, and diffusion restriction, suggesting that mMRE‐derived biomechanical signatures can capture imaging‐accessible manifestations of fundamental tumor biology during progression [39].

This study has several limitations. The orthotopic VX2 model represents liver metastases of an anaplastic squamous cell carcinoma and may not fully capture the biological and stromal heterogeneity of primary human liver cancers such as HCC. Nevertheless, it reproduces key imaging features of hypervascularized tumors like HCC, offers reproducible growth kinetics, and enables longitudinal investigations on a clinical MRI platform, which was the primary translational objective of this study. Our findings also require validation in other liver pathologies, particularly in fibrotic or cirrhotic livers, as VX2 tumors developed in non‐fibrotic tissue and background fibrosis is known to alter liver biomechanics. In addition, tumor implantation is a mechanical perturbation of the liver that may transiently affect early mMRE measurements but is likely to diminish by the first imaging timepoint as measurable biomechanical alterations are confined to the tumor rather than the needle track. Finally, histologic and proteomic analyses were performed at a single time point, limiting assessment of the temporal relationship between ECM remodeling and in vivo biomechanical changes.

## 5 Conclusion

This study provides longitudinal in vivo evidence that mMRE can noninvasively track biomechanical changes during liver tumor progression using a clinical imaging platform. Tumor stiffness and viscosity increased early, preceding changes in diffusion‐weighted imaging, and correlated with stromal remodeling assessed by histology and proteomics. These findings support the concept of a biomechanical tumor niche as an imaging‐accessible hallmark of liver cancer and suggest tumor stiffness and viscosity as imaging biomarkers reflecting dynamic interactions of the TME during carcinogenesis. By enabling noninvasive, longitudinal assessment of these biomechanical signatures in vivo, mMRE offers a translational framework with the potential to advance liver cancer imaging toward earlier detection, biologically informed risk stratification, and personalized monitoring of patients with or at risk for liver cancer.

## Author Contributions

*Conceptualization*: **Lynn Jeanette Savic**, **Jing Guo**. *Data curation*: **Justus Ramtke**, **Friederike Hesse**, **Lasse Noack**, **Justus Klockner**, **Yubei He**, **Yanglei Wu**, **Selma Saclier**, **Robin Schmidt**, **Richard Ruppel**, Lynn Jeanette Savic. *Formal analysis*: Justus Ramtke, Friederike Hesse, Justus Klockner, **Yu Liu**, **Nikolaus Berndt**. *Funding acquisition*: Lynn Jeanette Savic, Jing Guo, **Ingolf Sack**, Nikolaus Berndt. *Investigation*: Justus Ramtke, Friederike Hesse, Lasse Noack, Justus Klockner, Yubei He, Yu Liu, Selma Saclier, Salma A.S. Abosabie, **Michael Mülleder**, **Eyk Schellenberger**, Lynn Jeanette Savic. *Methodology*: Lynn Jeanette Savic, Justus Ramtke, Friederike Hesse, Jing Guo. *Project administration*: Lynn Jeanette Savic, Jing Guo, Ingolf Sack. *Resources*: Lynn Jeanette Savic, Michael Mülleder, Eyk Schellenberger, **Nicola Beindorff**, **Dominik N. Müller**, Jing Guo, Ingolf Sack. *Software*: **Tom Meyer**, Yanglei Wu, Robin Schmidt, Richard Ruppel. *Supervision*: Lynn Jeanette Savic, Jing Guo, Ingolf Sack, Dominik N. Müller. *Validation*: Justus Ramtke, Friederike Hesse, Lynn Jeanette Savic, **Shraga Nahum Goldberg**. *Visualization*: Justus Ramtke, Shraga Nahum Goldberg, Lynn Jeanette Savic. *Writing* – *original draft*: Justus Ramtke. *Writing* – *review & editing*: All authors.

## Funding

This work was funded by the Deutsche Forschungsgemeinschaft (DFG, German Research Foundation) through the research unit FOR5628 (Project‐ID 513752256, projects B02 and A02) and GRK2260 BIOQIC.

## Ethics Statement

The Berlin State Office for Health and Social Affairs (LAGeSo) approved the Animal Experiments under the Registry Number G150/20. Experiments were conducted in accordance with the Federation of European Laboratory Animal Science Associations (FELASA) Regulations.

## Conflicts of Interest

The authors declare no conflicts of interest.

## Supporting information




**Supporting File 1**: advs77626‐sup‐0001‐SuppMat.tif.

**Supporting File 2**: advs77626‐sup‐0002‐SuppMat.docx.

## Data Availability

MRE imaging data were post‐processed and stored using the following platform: https://bioqic‐apps.charite.de/. Protein‐protein interaction analysis can be viewed by STRING‐dB (https://string‐db.org/) using the protein names provided in Table S1. Pathway enrichment analysis can be viewed by Enrichr (https://maayanlab.cloud/Enrichr/) using the protein names provided in Table S1. The data that support the findings of this study are available from the corresponding author upon reasonable request.
