# Supplementary material for: Longitudinal Multifrequency MR Elastography Reveals Biomechanical Signatures of Progression in a Translational Liver Tumor Model
Source: Adv Sci (Weinh). 2026 Sep 16:e77626. Online ahead of print. doi: 10.1002/advs.77626 (PMC13583118; doi:10.1002/advs.77626)
Supplement: Supplementary file 1 [file ADVS-9999-e77626-s001.docx]

**Fig. S1:** **Liver and tumor segmentations.** Representative 3D segmentation masks of liver (left) and tumor (right) over a time period of five growth scans.

**Tab. S1:** Seven protein clusters derived from STRING-dB k-means clustering

| **Cluster number/color** | **Gene count** | **Primary description** | **Secondary description** | **Tertiary description** | **Protein names** |
| --- | --- | --- | --- | --- | --- |
| 1/red | 13 | Integrin-mediated signaling pathway | Leukocyte transendothelial migration | - | ITGB2, GSN, ACTN1, PFN1, TLN1, ITGB4, ITGAM, ITGA4, LPXN, MYH10, THY1, LAMB1, TES |
| 2/brown | 12 | Collagen fibril organization | - | - | COL5A2, BGN, BMP1, COL3A1, SPARC, TGFB2, TGFBR1, DAB2, TGFBR3, LUM, DPT, PPIC |
| 3/olive | 8 | Protein hydroxylation | Collagen biosynthesis and modifying enzymes | - | FKBP10, P3H1, PLOD1, P4HA1, COL15A1, P3H3, PLOD2, ERO1A |
| 4/green | 7 | Hyaluronan uptake and degradation | Peroxidasin, peroxidase domain, and NADE modulates death signalling | Von Willebrand factor (vWF) type C domain | PXDN, CD44, EZR, PALLD, PXDNL, SLC9A1, SDCBP |
| 5/blue | 5 | Activation of Matrix Metalloproteinases | - | - | PLAUR, CTSK, TIMP1, MMP13, MMP8 |
| 6/light sky blue | 3 | Angiogenesis involved in coronary vascular morphogenesis | Vascular endothelial growth factor binding | SEMA3A-Plexin repulsion signaling by inhibiting Integrin adhesion | FYN, PDGFRB, NRP1 |
| 7/medium blue | 2 | Heparin biosynthesis | - | - | B4GALT7, EXT1 |

**Tab. S2:** Biological Process, Molecular Function, Cellular Component, KEGG, and Reactome pathway enrichment of STRING-dB derived clusters.

| **Cluster** | **Database** | **Term ID** | **Term** | **Strength** | **Signal** | **False discovery rate (FDR)** |
| --- | --- | --- | --- | --- | --- | --- |
| 1 Integrin-mediated adhesion & cytoskeletal remodeling | Biological Process | [GO:0007229](http://amigo.geneontology.org/amigo/term/GO:0007229) | Integrin-mediated signaling pathway | 2.09 | 3.41 | 2.36e-08 |
|  | Molecular Function | [GO:0005178](http://amigo.geneontology.org/amigo/term/GO:0005178) | Integrin binding | 2.05 | 4.59 | 4.75e-12 |
|  | Cellular Component | [GO:0005925](http://amigo.geneontology.org/amigo/term/GO:0005925) | Focal adhesion | 1.82 | 2.73 | 1.81e-07 |
|  | KEGG Pathways | [ocu04810](https://www.kegg.jp/kegg-bin/show_pathway?ocu04810) | Regulation of actin cytoskeleton | 1.83 | 3.38 | 1.31e-09 |
|  | Reactome Pathways | [MAP-1474244](https://reactome.org/content/query?q=R-*-1474244) | Extracellular matrix organization | 1.37 | 1.04 | 0.0024 |
| 2 Collagen fibril organization | Biological Process | [GO:0030199](http://amigo.geneontology.org/amigo/term/GO:0030199) | Collagen fibril organization | 2.35 | 4.20 | 2.08e-09 |
|  | Molecular Function | [GO:0005201](http://amigo.geneontology.org/amigo/term/GO:0005201) | Extracellular matrix structural constituent | 1.80 | 0.97 | 0.0111 |
|  | Cellular Component | [GO:0062023](http://amigo.geneontology.org/amigo/term/GO:0062023) | Collagen-containing extracellular matrix | 1.65 | 2.30 | 9.81e-07 |
|  | KEGG Pathways | - | - | - | - | - |
|  | Reactome Pathways | [MAP-1650814](https://reactome.org/content/query?q=R-*-1650814) | Collagen biosynthesis and modifying enzymes | 1.85 | 1.78 | 0.00015 |
| 3 Collagen hydroxylation & maturation | Biological Process | [GO:0018126](http://amigo.geneontology.org/amigo/term/GO:0018126) | Protein hydroxylation | 2.79 | 8.29 | 2.53e-15 |
|  | Molecular Function | [GO:0019798](http://amigo.geneontology.org/amigo/term/GO:0019798) | Procollagen-proline dioxygenase activity | 3.04 | 3.52 | 1.62e-06 |
|  | Cellular Component | [GO:0005783](http://amigo.geneontology.org/amigo/term/GO:0005783) | Endoplasmic reticulum | 1.06 | 1.02 | 0.00019 |
|  | KEGG Pathways | - | - | - | - | - |
|  | Reactome Pathways | [MAP-1474290](https://reactome.org/content/query?q=R-*-1474290) | Collagen formation | 2.13 | 4.47 | 8.11e-10 |
| 4 Hyaluronan/GAG remodeling | Biological Process | - | - | - | - | - |
|  | Molecular Function | - | - | - | - | - |
|  | Cellular Component | - | - | - | - | - |
|  | KEGG Pathways | - | - | - | - | - |
|  | Reactome Pathways | [MAP-2160916](https://reactome.org/content/query?q=R-*-2160916) | Hyaluronan uptake and degradation | 2.54 | 0.98 | 0.0287 |
| 5 ECM/collagen degradation | Biological Process | [GO:0030574](http://amigo.geneontology.org/amigo/term/GO:0030574) | Collagen catabolic process | 2.61 | 2.34 | 0.00044 |
|  | Molecular Function | - | - | - | - | - |
|  | Cellular Component | - | - | - | - | - |
|  | KEGG Pathways | - | - | - | - | - |
|  | Reactome Pathways | [MAP-1592389](https://reactome.org/content/query?q=R-*-1592389) | Activation of Matrix Metalloproteinases | 2.70 | 4.84 | 7.47e-08 |

**Tab. S3:** Pathway enrichment in Reactome Pathways 2024

| **Name** | **Odds Ratio** | **Combined score** | **Adjusted *P*-value** | **-Log10(Adjusted *P*-value)** | **Category** |
| --- | --- | --- | --- | --- | --- |
| Extracellular Matrix Organization | 71.55 | 5285.16 | 2.36E-30 | 29.627088 | ECM remodeling & degradation |
| Collagen Formation | 80.45 | 3236.09 | 4.84E-16 | 15.3148855 | Collagen synthesis & maturation |
| Collagen Biosynthesis and Modifying Enzymes | 75.29 | 2282.26 | 6.49E-12 | 11.1878892 | Collagen synthesis & maturation |
| Degradation of the Extracellular Matrix | 38.12 | 1034.07 | 1.18E-10 | 9.92959268 | ECM remodeling & degradation |
| Assembly of Collagen Fibrils and Other Multimeric Structures | 59.98 | 1354.75 | 8.84E-09 | 8.05349861 | Collagen synthesis & maturation |
| ECM Proteoglycans | 46.9 | 985.17 | 3.59E-08 | 7.44502654 | Proteoglycan & GAG biology |
| Integrin Cell Surface Interactions | 41.47 | 838.03 | 6.83E-08 | 7.16564289 | Cell–matrix adhesion & cytoskeletal remodeling |
| Collagen Degradation | 46.77 | 852.89 | 4.28E-07 | 6.36835334 | ECM remodeling & degradation |
| Hemostasis | 8.75 | 139.23 | 3.885E-06 | 5.41060898 | Vascular remodeling & angiogenesis |
| Non-integrin membrane-ECM Interactions | 40.94 | 607.46 | 0.00001025 | 4.98927613 | Cell–matrix adhesion & cytoskeletal remodeling |
| Syndecan Interactions | 75.34 | 1081.72 | 0.00001506 | 4.82217503 | Proteoglycan & GAG biology |
| Glycosaminoglycan Metabolism | 22.35 | 315.59 | 0.0000155 | 4.8096683 | Proteoglycan & GAG biology |
| Axon Guidance | 9.14 | 128.95 | 0.0000155 | 4.8096683 | Cell–matrix adhesion & cytoskeletal remodeling |
| Platelet Degranulation | 22.16 | 311.96 | 0.0000155 | 4.8096683 | Vascular remodeling & angiogenesis |
| TGFBR3 Regulates TGF-beta Signaling | 254.62 | 3569.43 | 0.0000155 | 4.8096683 | Stromal & growth-factor signaling |
| Response to Elevated Platelet Cytosolic Ca2+ | 21.28 | 294.82 | 0.00001719 | 4.76472412 | Vascular remodeling & angiogenesis |
| Nervous System Development | 8.7 | 119.06 | 0.00001922 | 4.71624662 | Other / nonspecific signaling |
| Activation of Matrix Metalloproteinases | 59.73 | 807.75 | 0.00002122 | 4.67325462 | ECM remodeling & degradation |
| Diseases Associated With Glycosaminoglycan Metabolism | 46.8 | 591.12 | 0.00004903 | 4.30953811 | Proteoglycan & GAG biology |
| Platelet Activation, Signaling and Aggregation | 12.57 | 157.01 | 0.00005377 | 4.26945996 | Vascular remodeling & angiogenesis |
| SEMA3A-Plexin Repulsion Signaling by Inhibiting Integrin Adhesion | 115.7 | 1406.63 | 0.00007123 | 4.14733706 | Cell–matrix adhesion & cytoskeletal remodeling |
| Signaling by TGFBR3 | 38.46 | 458.01 | 0.0000873 | 4.05898576 | Stromal & growth-factor signaling |
| Semaphorin Interactions | 28.83 | 312.39 | 0.0002436 | 3.61332272 | Cell–matrix adhesion & cytoskeletal remodeling |
| Signaling by Receptor Tyrosine Kinases | 7.01 | 69.34 | 0.0005977 | 3.22351674 | Stromal & growth-factor signaling |
| MET Activates PTK2 Signaling | 47.1 | 460.35 | 0.0006488 | 3.18788916 | Cell–matrix adhesion & cytoskeletal remodeling |
| Metabolism of Carbohydrates | 9.44 | 88.84 | 0.0008994 | 3.04604712 | Other / nonspecific signaling |
| MET Promotes Cell Motility | 33.45 | 295.27 | 0.001547 | 2.81050969 | Cell–matrix adhesion & cytoskeletal remodeling |
| Neutrophil Degranulation | 6.73 | 58.24 | 0.00178 | 2.74958 | Immune & inflammatory signaling |
| Collagen Chain Trimerization | 30.99 | 267.06 | 0.00178 | 2.74958 | Collagen synthesis & maturation |
| TGF-beta Receptor Signaling Activates SMADs | 28.88 | 243.12 | 0.002096 | 2.67860872 | Stromal & growth-factor signaling |
| L1CAM Interactions | 14.87 | 124.6 | 0.002108 | 2.67612939 | Cell–matrix adhesion & cytoskeletal remodeling |
| Cell Surface Interactions at the Vascular Wall | 9.61 | 78.48 | 0.002531 | 2.59670785 | Vascular remodeling & angiogenesis |
| TGFBR3 PTM Regulation | 92.32 | 739.5 | 0.002868 | 2.54242085 | Stromal & growth-factor signaling |
| Heparan Sulfate Heparin (HS-GAG) Metabolism | 23.52 | 184.53 | 0.00324 | 2.48945499 | Proteoglycan & GAG biology |
| Hyaluronan Uptake and Degradation | 83.08 | 650.5 | 0.00324 | 2.48945499 | Proteoglycan & GAG biology |
| Signaling by PDGF | 23.09 | 179.98 | 0.00326 | 2.4867824 | Stromal & growth-factor signaling |
| Diseases of Glycosylation | 12.13 | 92.6 | 0.003724 | 2.42899033 | Proteoglycan & GAG biology |
| NCAM Signaling for Neurite Out-Growth | 21.16 | 159.79 | 0.00394 | 2.40450378 | Cell–matrix adhesion & cytoskeletal remodeling |
| Signaling by TGFB Family Members | 10.96 | 79.66 | 0.004996 | 2.30137757 | Stromal & growth-factor signaling |
| Sema3A PAK Dependent Axon Repulsion | 59.33 | 429.46 | 0.004996 | 2.30137757 | Cell–matrix adhesion & cytoskeletal remodeling |
| CRMPs in Sema3A Signaling | 59.33 | 429.46 | 0.004996 | 2.30137757 | Cell–matrix adhesion & cytoskeletal remodeling |
| Hyaluronan Metabolism | 55.38 | 393.96 | 0.005519 | 2.25813961 | Proteoglycan & GAG biology |
| Crosslinking of Collagen Fibrils | 51.91 | 363.29 | 0.006054 | 2.21795758 | Collagen synthesis & maturation |
| Signaling by MET | 16.69 | 115.02 | 0.006456 | 2.19003648 | Stromal & growth-factor signaling |
| Ephrin Signaling | 48.86 | 336.55 | 0.006456 | 2.19003648 | Cell–matrix adhesion & cytoskeletal remodeling |
| Defective B4GALT7 Causes EDS, Progeroid Type | 46.14 | 313.05 | 0.007006 | 2.15452987 | Proteoglycan & GAG biology |
| Signal Transduction | 2.86 | 18.95 | 0.008082 | 2.09248115 | Other / nonspecific signaling |
| Nephrin Family Interactions | 39.54 | 257.15 | 0.008897 | 2.05075641 | Cell–matrix adhesion & cytoskeletal remodeling |
| EPH-Ephrin Signaling | 14.24 | 91.89 | 0.009183 | 2.03701542 | Cell–matrix adhesion & cytoskeletal remodeling |
| Signaling by TGF-beta Receptor Complex | 13.93 | 89 | 0.009572 | 2.01899731 | Stromal & growth-factor signaling |
| A Tetrasaccharide Linker Sequence Is Required for GAG Synthesis | 34.59 | 216.48 | 0.01059 | 1.97510404 | Proteoglycan & GAG biology |
| Innate Immune System | 3.62 | 22.58 | 0.01059 | 1.97510404 | Immune & inflammatory signaling |
| Developmental Biology | 3.38 | 21.04 | 0.01059 | 1.97510404 | Other / nonspecific signaling |
| EPHA-mediated Growth Cone Collapse | 30.75 | 185.7 | 0.01257 | 1.90066472 | Cell–matrix adhesion & cytoskeletal remodeling |
| Laminin Interactions | 29.65 | 177.06 | 0.0132 | 1.87942607 | Cell–matrix adhesion & cytoskeletal remodeling |
| Interleukin-4 and Interleukin-13 Signaling | 11.62 | 68.43 | 0.01408 | 1.85139735 | Immune & inflammatory signaling |
| Diseases of Metabolism | 6.59 | 35.97 | 0.02122 | 1.67325462 | Other / nonspecific signaling |
| NCAM1 Interactions | 20.74 | 110.1 | 0.02431 | 1.61421504 | Cell–matrix adhesion & cytoskeletal remodeling |
| Netrin-1 Signaling | 19.75 | 103.05 | 0.02618 | 1.58203036 | Cell–matrix adhesion & cytoskeletal remodeling |
| Immune System | 2.63 | 13.58 | 0.0272 | 1.5654311 | Immune & inflammatory signaling |
| Chondroitin Sulfate Dermatan Sulfate Metabolism | 17.28 | 85.84 | 0.03247 | 1.48851771 | Proteoglycan & GAG biology |
| Cell-Cell Communication | 8.21 | 40.59 | 0.03264 | 1.48624985 | Cell–matrix adhesion & cytoskeletal remodeling |
| Sensory Processing of Sound by Outer Hair Cells of the Cochlea | 15.64 | 74.84 | 0.03781 | 1.42239332 | Other / nonspecific signaling |
| Toll-like Receptor Cascades | 7.38 | 34.45 | 0.04187 | 1.37809704 | Immune & inflammatory signaling |

**Tab. S4:** Pathway enrichment in WikiPathways Human 2024

| **Name** | **Odds Ratio** | **Combined score** | **Adjusted**  ***P*-value** | **-Log10(Adjusted**  ***P*-value)** | **Category** |
| --- | --- | --- | --- | --- | --- |
| Type I Collagen Synthesis Osteogenesis Imperfecta WP4786 | 100.62 | 2253.23 | 2.99E-08 | 7.52389328 | Collagen synthesis & maturation |
| Focal Adhesion WP306 | 19.7 | 340.48 | 0.00000249 | 5.60380065 | Cell–matrix adhesion & cytoskeletal remodeling |
| Regulation Of Actin Cytoskeleton WP51 | 22.55 | 366.21 | 4.686E-06 | 5.32919772 | Cell–matrix adhesion & cytoskeletal remodeling |
| Integrin Mediated Cell Adhesion WP185 | 28.5 | 441.15 | 7.532E-06 | 5.12308969 | Cell–matrix adhesion & cytoskeletal remodeling |
| 11P11 2 Copy Number Variation Syndrome WP5348 | 43.35 | 654.77 | 8.774E-06 | 5.05680237 | Other / nonspecific signaling |
| Hippo Merlin Signaling Dysregulation WP4541 | 23.73 | 343.01 | 0.00001396 | 4.85511458 | Cell–matrix adhesion & cytoskeletal remodeling |
| Focal Adhesion PI3K Akt mTOR Signaling WP3932 | 12.69 | 178.46 | 0.00001776 | 4.75055704 | Cell–matrix adhesion & cytoskeletal remodeling |
| Metabolic Pathways Of Fibroblasts WP5312 | 61.87 | 844.52 | 0.00002345 | 4.62985715 | Stromal & growth-factor signaling |
| TGFB Smad Signaling WP5382 | 141.43 | 1790.11 | 0.00005624 | 4.24995469 | Stromal & growth-factor signaling |
| NRP1 Triggered Signaling In Pancreatic Cancer WP5144 | 35.32 | 409.37 | 0.000147 | 3.83268267 | Stromal & growth-factor signaling |
| Primary Focal Segmental Glomerulosclerosis FSGS WP2572 | 25.42 | 263.65 | 0.0004534 | 3.34351848 | Cell–matrix adhesion & cytoskeletal remodeling |
| Extracellular Vesicle Mediated Signaling In Recipient Cells WP2870 | 48.91 | 483.14 | 0.0006799 | 3.16755496 | Other / nonspecific signaling |
| Matrix Metalloproteinases WP129 | 47.1 | 460.35 | 0.0006961 | 3.15732837 | ECM remodeling & degradation |
| Pleural Mesothelioma WP5087 | 7.39 | 68.02 | 0.001142 | 2.9423339 | Other / nonspecific signaling |
| TGF Beta Signaling Pathway WP366 | 13.47 | 107.95 | 0.003498 | 2.45618019 | Stromal & growth-factor signaling |
| TYROBP Causal Network In Microglia WP3945 | 21.52 | 163.52 | 0.00498 | 2.30277066 | Immune & inflammatory signaling |
| Osteoclast Signaling WP12 | 59.33 | 429.46 | 0.006052 | 2.21810108 | Cell–matrix adhesion & cytoskeletal remodeling |
| Canonical And Non Canonical TGF B Signaling WP3874 | 55.38 | 393.96 | 0.006052 | 2.21810108 | Stromal & growth-factor signaling |
| Cells And Molecules Involved In Acute Inflammatory Response WP4493 | 55.38 | 393.96 | 0.006052 | 2.21810108 | Immune & inflammatory signaling |
| miR 509 3P Alteration Of YAP1 ECM Axis WP3967 | 55.38 | 393.96 | 0.006052 | 2.21810108 | Cell–matrix adhesion & cytoskeletal remodeling |
| Arrhythmogenic Right Ventricular Cardiomyopathy WP2118 | 17.87 | 126.54 | 0.006052 | 2.21810108 | Cell–matrix adhesion & cytoskeletal remodeling |
| Burn Wound Healing WP5055 | 17.87 | 126.54 | 0.006052 | 2.21810108 | ECM remodeling & degradation |
| Proteoglycan Biosynthesis WP4784 | 51.91 | 363.29 | 0.006052 | 2.21810108 | Proteoglycan & GAG biology |
| Congenital Generalized Lipodystrophy WP5101 | 51.91 | 363.29 | 0.006052 | 2.21810108 | Other / nonspecific signaling |
| Clear Cell Renal Cell Carcinoma Pathways WP4018 | 15.47 | 103.29 | 0.007995 | 2.09718153 | Other / nonspecific signaling |
| miRNA Targets In ECM And Membrane Receptors WP2911 | 41.52 | 273.73 | 0.00838 | 2.07675598 | Cell–matrix adhesion & cytoskeletal remodeling |
| Hypothesized Pathways In Pathogenesis Of Cardiovascular Disease WP3668 | 36.1 | 228.73 | 0.01043 | 1.98171569 | Vascular remodeling & angiogenesis |
| Complement System In Neuronal Development And Plasticity WP5090 | 12.3 | 74.36 | 0.01344 | 1.87160073 | Immune & inflammatory signaling |
| Inflammatory Response Pathway WP453 | 29.65 | 177.06 | 0.01397 | 1.85480359 | Immune & inflammatory signaling |
| Gastrin Signaling WP4659 | 11.31 | 65.75 | 0.01579 | 1.80161787 | Other / nonspecific signaling |
| Alpha 6 Beta 4 Signaling WP244 | 26.77 | 154.84 | 0.01579 | 1.80161787 | Cell–matrix adhesion & cytoskeletal remodeling |
| Type 2 Papillary Renal Cell Carcinoma WP4241 | 25.93 | 148.46 | 0.01622 | 1.78994915 | Other / nonspecific signaling |
| P38 MAPK Signaling WP400 | 25.15 | 142.52 | 0.01666 | 1.778325 | Other / nonspecific signaling |
| Neovascularization Processes WP4331 | 23.71 | 131.76 | 0.01804 | 1.74376347 | Vascular remodeling & angiogenesis |
| Ebola Virus Infection In Host WP4217 | 10.04 | 55.16 | 0.01871 | 1.72792621 | Immune & inflammatory signaling |
| VEGFA VEGFR2 Signaling WP3888 | 5.1 | 27.82 | 0.01897 | 1.72193267 | Vascular remodeling & angiogenesis |
| Microglia Pathogen Phagocytosis Pathway WP3937 | 21.83 | 118 | 0.01932 | 1.71399288 | Immune & inflammatory signaling |
| Glycosaminoglycan Synthesis In Fibroblasts WP5395 | 21.27 | 113.94 | 0.01975 | 1.7044329 | Proteoglycan & GAG biology |
| Neuroinflammation And Glutamatergic Signaling WP5083 | 9.23 | 48.59 | 0.02059 | 1.68634365 | Immune & inflammatory signaling |
| Fibrin Complement Receptor 3 Signaling WP4136 | 20.23 | 106.48 | 0.02059 | 1.68634365 | Immune & inflammatory signaling |
| TROP2 Regulatory Signaling WP5300 | 19.29 | 99.81 | 0.02196 | 1.65836766 | Other / nonspecific signaling |
| Chronic Hyperglycemia Impairment Of Neuron Function WP5283 | 18.85 | 96.72 | 0.02237 | 1.65033402 | Other / nonspecific signaling |
| Mechanoreg And Pathology Of YAP TAZ Hippo And Non Hippo Mechs WP4534 | 18.43 | 93.8 | 0.02279 | 1.64225567 | Cell–matrix adhesion & cytoskeletal remodeling |
| Hepatitis C And Hepatocellular Carcinoma WP3646 | 17.64 | 88.37 | 0.02415 | 1.61708286 | Immune & inflammatory signaling |
| Osteoarthritic Chondrocyte Hypertrophy WP5373 | 17.28 | 85.84 | 0.02456 | 1.60977164 | ECM remodeling & degradation |
| Epithelial To Mesenchymal Transition In Colorectal Cancer WP4239 | 8.05 | 39.4 | 0.02537 | 1.59567953 | Stromal & growth-factor signaling |
| Netrin UNC5B Signaling WP4747 | 16.58 | 81.14 | 0.02537 | 1.59567953 | Cell–matrix adhesion & cytoskeletal remodeling |
| Cardiac Progenitor Differentiation WP2406 | 16.26 | 78.95 | 0.02578 | 1.58871709 | Other / nonspecific signaling |
| TGF Beta Receptor Signaling WP560 | 15.94 | 76.85 | 0.02606 | 1.58402559 | Stromal & growth-factor signaling |
| Overlap Between Signal Transduction Pathways LMNA Laminopathies WP4879 | 15.64 | 74.84 | 0.02606 | 1.58402559 | Other / nonspecific signaling |
| Pathogenic Escherichia Coli Infection WP2272 | 15.64 | 74.84 | 0.02606 | 1.58402559 | Immune & inflammatory signaling |
| TGF Beta Receptor Signaling In Skeletal Dysplasias WP4816 | 14.8 | 69.3 | 0.02832 | 1.54790675 | Stromal & growth-factor signaling |
| PI3K Akt Signaling WP4172 | 5.11 | 23.51 | 0.03006 | 1.52201102 | Other / nonspecific signaling |
| Endochondral Ossification WP474 | 13.59 | 61.45 | 0.03138 | 1.50334706 | Collagen synthesis & maturation |
| Endochondral Ossification With Skeletal Dysplasias WP4808 | 13.59 | 61.45 | 0.03138 | 1.50334706 | Collagen synthesis & maturation |
| AGE RAGE Pathway WP2324 | 12.95 | 57.41 | 0.03369 | 1.47249899 | Other / nonspecific signaling |
| SARS CoV 2 Altering Angiogenesis Via NRP1 WP5065 | 101.77 | 446.44 | 0.0347 | 1.45967053 | Vascular remodeling & angiogenesis |
| Axon Guidance WP5289 | 11.83 | 50.51 | 0.03839 | 1.41578189 | Cell–matrix adhesion & cytoskeletal remodeling |
| Chromosomal And Microsatellite Instability In Colorectal Cancer WP4216 | 11.67 | 49.49 | 0.03874 | 1.41184038 | Other / nonspecific signaling |
| LDLRAD4 And What We Know About It WP4904 | 67.84 | 274.94 | 0.04604 | 1.33686469 | Other / nonspecific signaling |
| Acute Viral Myocarditis WP4298 | 9.97 | 39.44 | 0.04995 | 1.30146451 | Immune & inflammatory signaling |

**Tab. S5:** Pathway enrichment in KEGG 2026

| **Name** | **Odds Ratio** | **Combined score** | **Adjusted *P*-value** | **-log10(Adjusted *P*-value)** | **Category** |
| --- | --- | --- | --- | --- | --- |
| REGULATION OF ACTIN CYTOSKELETON | 28.85 | 832.05 | 3.583E-11 | 10.4457532 | Cell–matrix adhesion & cytoskeletal remodeling |
| INTEGRIN SIGNALING | 30.19 | 685.98 | 8.143E-09 | 8.08921557 | Cell–matrix adhesion & cytoskeletal remodeling |
| LEUKOCYTE TRANSENDOTHELIAL MIGRATION | 29.63 | 533.94 | 5.978E-07 | 6.22344409 | Immune & inflammatory signaling |
| FOCAL ADHESION | 19.7 | 340.48 | 9.395E-07 | 6.02710322 | Cell–matrix adhesion & cytoskeletal remodeling |
| IGSF CAM SIGNALING | 15.15 | 257.31 | 1.006E-06 | 5.99740202 | Cell–matrix adhesion & cytoskeletal remodeling |
| AMOEBIASIS | 28.8 | 447.58 | 3.571E-06 | 5.44721015 | Immune & inflammatory signaling |
| PROTEOGLYCANS IN CANCER | 16.93 | 243.59 | 9.674E-06 | 5.01439392 | Proteoglycan & GAG biology |
| CYTOSKELETON IN MUSCLE CELLS | 14.4 | 192.25 | 0.00002389 | 4.62178385 | Cell–matrix adhesion & cytoskeletal remodeling |
| LEISHMANIASIS | 24.01 | 243.82 | 0.0005178 | 3.28583795 | Immune & inflammatory signaling |
| ECM-RECEPTOR INTERACTION | 20.57 | 197.03 | 0.0008285 | 3.08170749 | Cell–matrix adhesion & cytoskeletal remodeling |
| RAP1 SIGNALING PATHWAY | 10.7 | 92.54 | 0.001915 | 2.71783122 | Cell–matrix adhesion & cytoskeletal remodeling |
| HUMAN T-CELL LEUKEMIA VIRUS 1 INFECTION | 10.25 | 86.61 | 0.002134 | 2.67080558 | Immune & inflammatory signaling |
| SHIGELLOSIS | 9.24 | 73.86 | 0.002995 | 2.52360317 | Immune & inflammatory signaling |
| OSTEOCLAST DIFFERENTIATION | 13.26 | 105.52 | 0.002995 | 2.52360317 | Immune & inflammatory signaling |
| MICRORNAS IN CANCER | 11.03 | 80.44 | 0.005458 | 2.26296647 | Other / nonspecific signaling |
| HUMAN PAPILLOMAVIRUS INFECTION | 6.75 | 44.7 | 0.009489 | 2.02277955 | Immune & inflammatory signaling |
| COMPLEMENT AND COAGULATION CASCADES | 15.1 | 99.81 | 0.009489 | 2.02277955 | Vascular remodeling & angiogenesis |
| ADHERENS JUNCTION | 14.24 | 91.89 | 0.009793 | 2.00908425 | Cell–matrix adhesion & cytoskeletal remodeling |
| RHEUMATOID ARTHRITIS | 14.24 | 91.89 | 0.009793 | 2.00908425 | Immune & inflammatory signaling |
| GLYCOSAMINOGLYCAN BIOSYNTHESIS - HEPARAN SULFATE / HEPARIN | 37.74 | 242.23 | 0.009793 | 2.00908425 | Proteoglycan & GAG biology |
| HEMATOPOIETIC CELL LINEAGE | 13.48 | 84.93 | 0.01032 | 1.9863203 | Immune & inflammatory signaling |
| HYPERTROPHIC CARDIOMYOPATHY | 13.34 | 83.64 | 0.01032 | 1.9863203 | Other / nonspecific signaling |
| AGE-RAGE SIGNALING PATHWAY IN DIABETIC COMPLICATIONS | 13.06 | 81.16 | 0.01046 | 1.98046832 | Stromal & growth-factor signaling |
| PROTEIN DIGESTION AND ABSORPTION | 12.8 | 78.79 | 0.01047 | 1.98005332 | Collagen synthesis & maturation |
| DILATED CARDIOMYOPATHY | 12.67 | 77.64 | 0.01047 | 1.98005332 | Other / nonspecific signaling |
| PLATELET ACTIVATION | 10.37 | 57.89 | 0.01714 | 1.76598918 | Vascular remodeling & angiogenesis |
| RELAXIN SIGNALING PATHWAY | 10.29 | 57.19 | 0.01714 | 1.76598918 | Stromal & growth-factor signaling |
| CELL ADHESION MOLECULE (CAM) INTERACTION | 8.31 | 41.43 | 0.0278 | 1.5559552 | Cell–matrix adhesion & cytoskeletal remodeling |
| HIPPO SIGNALING PATHWAY | 8.31 | 41.43 | 0.0278 | 1.5559552 | Cell–matrix adhesion & cytoskeletal remodeling |
| MALARIA | 17.28 | 85.84 | 0.0278 | 1.5559552 | Immune & inflammatory signaling |
| LEGIONELLOSIS | 15.35 | 72.92 | 0.03257 | 1.48718224 | Immune & inflammatory signaling |
| TIGHT JUNCTION | 7.61 | 36.1 | 0.03257 | 1.48718224 | Cell–matrix adhesion & cytoskeletal remodeling |
| TUBERCULOSIS | 7.17 | 32.92 | 0.03692 | 1.43273831 | Immune & inflammatory signaling |
| LYSINE DEGRADATION | 13.59 | 61.45 | 0.03805 | 1.41964534 | Other / nonspecific signaling |
| VIRAL CARCINOGENESIS | 6.93 | 31.2 | 0.03805 | 1.41964534 | Other / nonspecific signaling |
| PI3K-AKT SIGNALING PATHWAY | 4.86 | 21.54 | 0.03944 | 1.40406309 | Stromal & growth-factor signaling |
| VIRAL MYOCARDITIS | 12.55 | 54.95 | 0.04074 | 1.38997898 | Immune & inflammatory signaling |
| PATHOGENIC ESCHERICHIA COLI INFECTION | 6.47 | 27.93 | 0.04205 | 1.376234 | Immune & inflammatory signaling |
| DIABETIC CARDIOMYOPATHY | 6.3 | 26.8 | 0.04313 | 1.36522054 | Other / nonspecific signaling |
| GASTRIC ACID SECRETION | 11.67 | 49.49 | 0.04313 | 1.36522054 | Other / nonspecific signaling |
| CHRONIC MYELOID LEUKEMIA | 11.19 | 46.62 | 0.04439 | 1.35271485 | Other / nonspecific signaling |
| PANCREATIC CANCER | 11.04 | 45.72 | 0.04439 | 1.35271485 | Other / nonspecific signaling |
| PERTUSSIS | 11.04 | 45.72 | 0.04439 | 1.35271485 | Immune & inflammatory signaling |
